# Growth Forms and Functional Guilds Distribution of Soil Fungi in Coastal Versus Inland Sites of Victoria Land, Antarctica

**DOI:** 10.3390/biology10040320

**Published:** 2021-04-11

**Authors:** Fabiana Canini, József Geml, Pietro Buzzini, Benedetta Turchetti, Silvano Onofri, Luigi Paolo D’Acqui, Caterina Ripa, Laura Zucconi

**Affiliations:** 1 Department of Ecological and Biological Sciences, University of Tuscia, 01100 Viterbo, Italy; onofri@unitus.it (S.O.); cripa@unitus.it (C.R.); zucconi@unitus.it (L.Z.); 2 Biodiversity Dynamics Research Group, Naturalis Biodiversity Center, 2300 RA Leiden, The Netherlands; jozsef.geml@naturalis.nl; 3 MTA-EKE Lendület Environmental Microbiome Research Group, Eszterházy Károly University, H-3300 Eger, Hungary; 4 Department of Agricultural, Food and Environmental Sciences, University of Perugia, 06121 Perugia, Italy; pietro.buzzini@unipg.it (P.B.); benedetta.turchetti@unipg.it (B.T.); 5 Research Institute of Terrestrial Ecosystems, National Research Council of Italy (IRET-CNR), 50019 Sesto Fiorentino, Italy; luigipaolo.dacqui@cnr.it

**Keywords:** growth forms, lifestyles, evenness

## Abstract

**Simple Summary:**

Antarctica represents one of the most limiting environments on Earth, where exposed soils, transiently present on coastal areas and permanently exposed on McMurdo Dry Valleys, are one of the few substrata supporting microbial communities. Within these communities, fungi are one of the dominant components and play pivotal roles in recycling the extremely limited organic matter available. Despite their astonishing adaptations, the diversity of these communities and the factors determining their resistance are still poorly known. In this optic, this study aimed to give insights in the environmental parameters determining the structure of these communities and their adaptive strategies, in terms of growth forms and lifestyles. We found that abiotic conditions were the main drivers of well differentiated communities. Additionally, we highlighted that most species seemed to be highly adapted to this habitat, which may result in a relatively low resilience ability to changes in environmental condition. Both these results are of particular concern in light of global warming. Even minimal changes to environmental conditions may dramatically alter the Antarctic soil communities, risking the disappearance of many species still undescribed.

**Abstract:**

In Victoria Land, Antarctica, ice-free areas are restricted to coastal regions and dominate the landscape of the McMurdo Dry Valleys. These two environments are subjected to different pressures that determine the establishment of highly adapted fungal communities. Within the kingdom of fungi, filamentous, yeasts and meristematic/microcolonial growth forms on one side and different lifestyles on the other side may be considered adaptive strategies of particular interest in the frame of Antarctic constraints. In this optic, soil fungal communities from both coastal and Dry Valleys sites, already characterized thorough ITS1 metabarcoding sequencing, have been compared to determine the different distribution of phyla, growth forms, and lifestyles. Though we did not find significant differences in the richness between the two environments, the communities were highly differentiated and Dry Valleys sites had a higher evenness compared to coastal ones. Additionally, the distribution of different growth forms and lifestyles were well differentiated, and their diversity and composition were likely influenced by soil abiotic parameters, among which soil granulometry, pH, P, and C contents were the potential main determinants.

## 1. Introduction

Although the Antarctic surface is nearly completely covered by ice, ice-free regions patchily occur across the continent, representing only 0.3% of the total area [1]. In continental Antarctica, they are restricted to coastal regions (as in Victoria, Princess Elizabeth, Mac Robertson, and Enderby Lands), to higher peaks of isolated mountains emerging from the ice cover (nunataks, along the Transantarctic, and the Prince Charles Mountains), and to the McMurdo Dry Valleys in South Victoria Land. These sites are characterized by different environmental conditions and offer a variety of soil environments, ranging from oligotrophic to copiotrophic, water-saturated to hyper-arid, and extremely cold to geothermally heated. The soil and mineral substrata have an important role in supporting the microbial colonization.

Since the beginning of the 20th century, Antarctica has attracted the interest of microbiologists, and many studies over the years focused on soil microbial diversity [2,3,4]. Starting from the pioneering culture-dependent studies dating back to the 1960s [5,6,7,8,9], a lot of research papers found a great number of cosmopolitan fungal species, mostly transported from other continents by wind and possibly sea birds, and others considered endemic. The introduction of molecular phylogenetic and metagenomic methods have given researchers a deeper understanding of fungal diversity, allowing them to identify a greater number of microorganisms than previously expected [10]. Geographic isolation and the selection pressure by the harsh environment may be the main drivers promoting speciation. Previously unknown species are frequently encountered, often with potential biotechnological interest. For example, unusual biochemical pathways have been reported from Antarctic strains, allowing them to generate new antimicrobial, herbicidal, and antitumor compounds [11,12,13,14,15].

In different studies focusing on soil fungi, Antarctic communities appeared highly specialized and mostly structured by abiotic factors due to limited biotic interactions [16,17,18,19,20,21]. Several authors reported the lack of a significant effect of latitude on fungal diversity [18,20,22,23,24], and the local environmental parameters have commonly been suggested as main drivers of fungal diversity in continental Antarctica [24,25]. Despite the studies published so far, Cowan et al. [25] highlighted that the drivers of soil microbial diversity at high latitudes are still scarcely known, with mean annual temperature and the secondary effects of temperature on water activity being the main candidates.

Victoria Land may be counted as the most studied area of continental Antarctica from the microbiological point of view [26] due to its diversity of habitats, such as coastal sites, nunataks, and the Dry Valleys in the south, all offering a great variety of terrestrial environments.

In coastal sites, seasonally ice-free areas, impacted by sea mammals and birds at some locations, have a relatively high water availability and are locally covered by a great diversity of organisms. Among these, bryophytes, lichens, cyanobacteria, and algal associations, as well as fungal and bacterial microorganisms associated with their thalli and present in the soil beneath them, make up biological soil crusts (BSCs) that are key components of polar environments. They are unique ecosystems with an unexpectedly large diversity of different autotrophic, heterotrophic, and saprotrophic organisms and can be regarded as oases in polar deserts [27]. Very few studies have been carried out on the composition and functionality of microbial communities beneath BSCs in continental Antarctica [20], despite their wide documented presence, even outside Victoria Land, such as in Vestfold Hills, Sør Rondane Mountains, Mac Robertson, Wilkes, and Enderby Lands [28,29]. The composition of the vegetation communities in continental Antarctica is suggested to be an indicator of climate change; considering the strong relationship between vegetation associations and soil microbial communities [30]; these latter may play a determinant role in the adaptive strategies and resilience abilities of vegetal communities.

More than anywhere else, the area of Dry Valleys of Southern Victoria Land is characterized by a combination of several stress factors, including extremely low temperatures, high solar and UV radiations during the austral summer, frequent freeze–thaw cycles even within a single day, extreme aridity, and local high salt concentration. The annual precipitation in snowfall is only 3–50 mm water equivalent, with the highest values nearest the coast and decreasing inland, making it one of the driest deserts on the planet [31]. The low water content of the soil is also due to the surrounding Transantarctic Mountains, which prevent the ice from the polar plateau flowing into the valleys, decrease the snowfall, and increase the sublimation rates [31]. Different types of soils characterize this area, depending on the distance from the coast, the inner lake shoreline or ephemeral water streams flowing at the valley floor, soil physicochemical parameters, or the presence and distance from colonized rocks in the surrounding area, all affecting soil fungal diversity. 

Within the kingdom of fungi, filamentous, yeasts, and meristematic/microcolonial growth forms may be considered different adaptive strategies, which are interesting in the frame of Antarctic constraints. Filamentous growth allows fungi to explore a greater surface in search of water and nutrients and makes it possible to form aggregates to retain water and nutrients in unconsolidated and sandy soils. However, unlike in the temperate zones where filamentous fungi prevail, yeasts are particularly well adapted to cold (polar and non-polar) environments, where they thrive combinations of several stressful conditions [32,33]. The success of soil-inhabiting yeasts is attributed to their structural and biochemical characteristics, e.g., the production of cold-adapted enzymes, cryoprotectant wall carbohydrates, and pigments, as well as higher amounts of polyunsaturated fatty acids in cytoplasmic membranes, a high amount of intracellular lipids, or the presence of capsules [32]. Black yeasts, also known as microcolonial or meristematic fungi, are a phylogenetically heterogeneous group of polyextremophilic fungi [34,35]. Under permanent stressful conditions, some black yeasts are able to shift between hyphal, yeast, and meristematic growth forms depending on the environment. The meristematic/microcolonial growth, forming black cauliflower-like colonies, often found in the interiors of rocks, provides them a greater resistance towards multiple stress factors. Additionally, their resistance is assured by the dense aggregation of melanized and thick-walled cells, encapsuled in a matrix of extracellular polymeric substances. They generally conclude their life cycle in a short time, not including the sexual, and in some taxa even asexual, reproduction [35,36].

In previous studies the total fungal diversity, composition, and functionality of the fungal communities associated with biological soil crusts from 18 sites in five coastal localities (coastal sites, CS) in Northern Victoria Land and one in Southern Victoria Land, namely, Botany Bay, with different stages of crust development, and from five sites in three localities along Taylor Valley in the McMurdo Dry Valleys (Taylor Valley sites, TVS) (South Victoria Land), have been identified and related to nutrient availability and physicochemical parameters [20,21]. In the present study, the taxonomic structure of these communities has been compared, as well as the differential distribution of filamentous fungi, yeasts and black yeasts, and of different lifestyles. The differences observed have been discussed in order to give some insights into the response of fungal diversity and adaptive strategies to different environmental conditions, namely presence and absence of above plant coverage and different soil physicochemical parameters.

## 2. Materials and Methods

### 2.1. Description of the Localities

**Figure 1:**
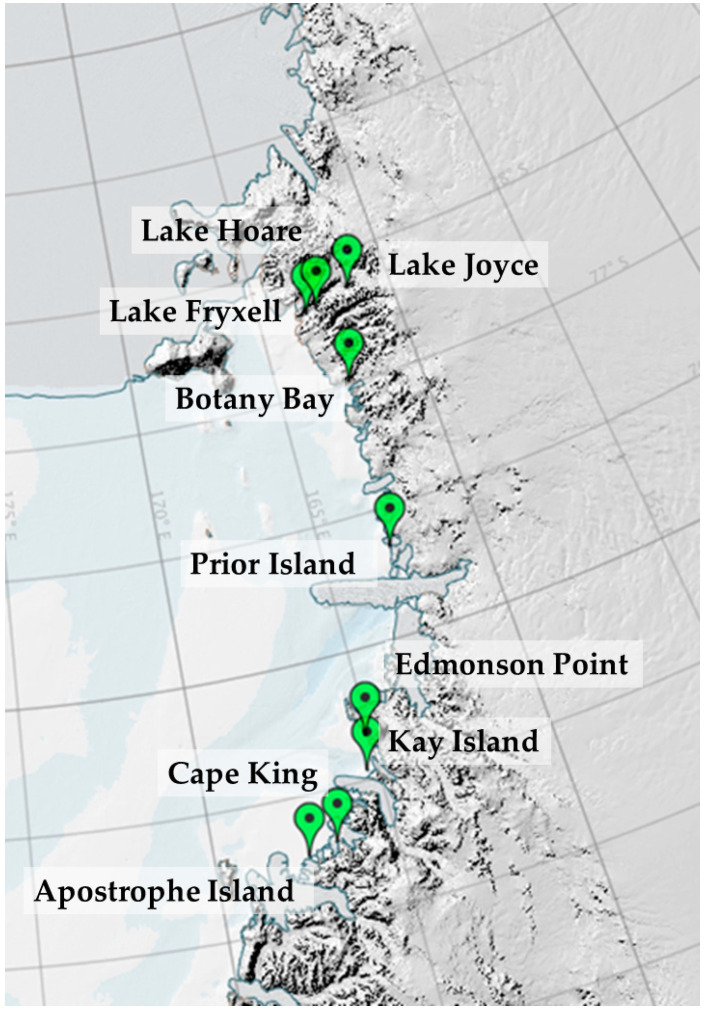
Map of sampling localities.

Three replicate soils samples were collected in 18 sites, and their fungal communities were already analyzed within two works dealing with Northern and Southern Victoria Land, respectively [20,21]. Thirteen sites were distributed in six localities, namely, Apostrophe Island (73°31′ S 167°25′ E), Cape King (73°35′ S 166°37′ E), Kay Island (74°04′ S 165°18′ E), Edmonson Point (74°19′ S 165°07′ E), Prior Island (75°40′ S 162°53′ E), and Botany Bay (77°00′ S 162°32′ E), which are located along the Ross Sea coast and cover a latitudinal gradient of 4° (coastal sites, CS). In these coastal sites, soil samples were collected just beneath the biological soil crust coverage (Figure 1) [20]. A further five sites from three localities were selected along the Taylor Valley (TVS, Taylor Valley sites) [21], a snow-free valley of the McMurdo area. Soil samples were collected close to three lakes, namely, Lake Fryxell (77°36′ S 163°16′ E), Lake Hoare (77°37′ S 162°53′ E), and Lake Joyce (77°42′ S 161°34′ E), but far enough away to not be influenced by the water ecosystem (Figure 1) [21].

### 2.2. Soil Physicochemical and Molecular Analyses

Soil physicochemical properties of both CS and TVS were determined and reported in Canini et al. [20,21] and used for statistical analyses. For the molecular analyses, the metagenomic DNA was extracted from each soil sample, amplified, and sequenced on an Illumina MiSeq platform. Demultiplexed sequences were then processed with the Amplicon ToolKit (AMPtk) for NGS data (formally UFITS) v.1.2.1 [37]. Starting reads were subjected to quality trimming and PhiX screening using USEARCH with default parameters (v. 9.2.64) [38]. Reads with less than 100 bp were removed, ones longer than 300 bp were trimmed, and paired-end reads were merged in one step. Individual sample sequence files were merged into a single file and clustered with a 97% identity threshold into operational taxonomic units (OTUs) using VSEARCH v 2.7.0 [39], simultaneously removing putative chimeras. Taxonomy was assigned to OTUs based on the curated UNITE+INSD reference database dynamic species hypotheses (UTAX release of 10 October 2017) using USEARCH (v. 9.2.64) [38]. Singletons (namely, OTUs with only one read in the dataset) and OTUs with less than 70% identity to a fungal species hypothesis (SH) were excluded for the following analyses. Functional assignment for OTUs with more than 90% similarity to a fungal SH with known ecological function were made by FunGuild [40] and manually checked [20,21]. Different growth form strategies were identified manually for genera with identities higher than 93% to known fungal genera.

### 2.3. Statistical Analyses

All analyses were carried out with the *vegan* package [41] in R [42]. Total fungal richness, richness of dominant phyla and classes, of different growth forms (i.e., filamentous fungi, yeasts, and black yeasts) and of the two main lifestyles (lichenized and saprotrophic fungi), their relative abundances, and their evenness (e^H/S^) were compared among the two environments using ANOVA and Tukey’s HSD test (significant for *p* < 0.05). Relationships between edaphic physicochemical parameters and total fungal richness, richness of dominant phyla and classes, of different growth forms and lifestyles, and their relative abundances were investigated through linear regression analyses (significant for *p* < 0.05; marginally significant for 0.05 < *p* < 0.1).

Non-metric multidimensional scaling (NMDS) was run on the Hellinger-transformed OTU table. Ordinations were run separately for the total community, the three growth forms, and the two main lifestyles with the following specifications: distance measure = Bray–Curtis, dimensions = 2, initial configurations = 100, model = global, maximum number of iterations = 200, and convergence ratio for stress = 0.999999. We used the *envfit* function to fit edaphic physicochemical parameters listed above onto the NMDS ordinations. In addition, we tested whether fungal communities were significantly (*p* < 0.01) different among different localities and among the two different environments using the multi-response permutation procedure (MRPP).

Permutational multivariate analysis of variance (PerMANOVA) [43] was carried out on Bray–Curtis distance matrices of Hellinger-transformed OTU tables with 9999 permutations, with the *adonis* function, in order to determine the effect of each soil physicochemical parameter on the observed variance of the total community, the dominant phyla, the growth forms, and the lifestyles. Finally, to account for correlations among environmental variables, we performed a forward selection of parameters based on the previous results, including only significant environmental variables (*p* < 0.05) in the final models.

## 3. Results

### 3.1. Taxonomic Characterization

**Figure 2:**
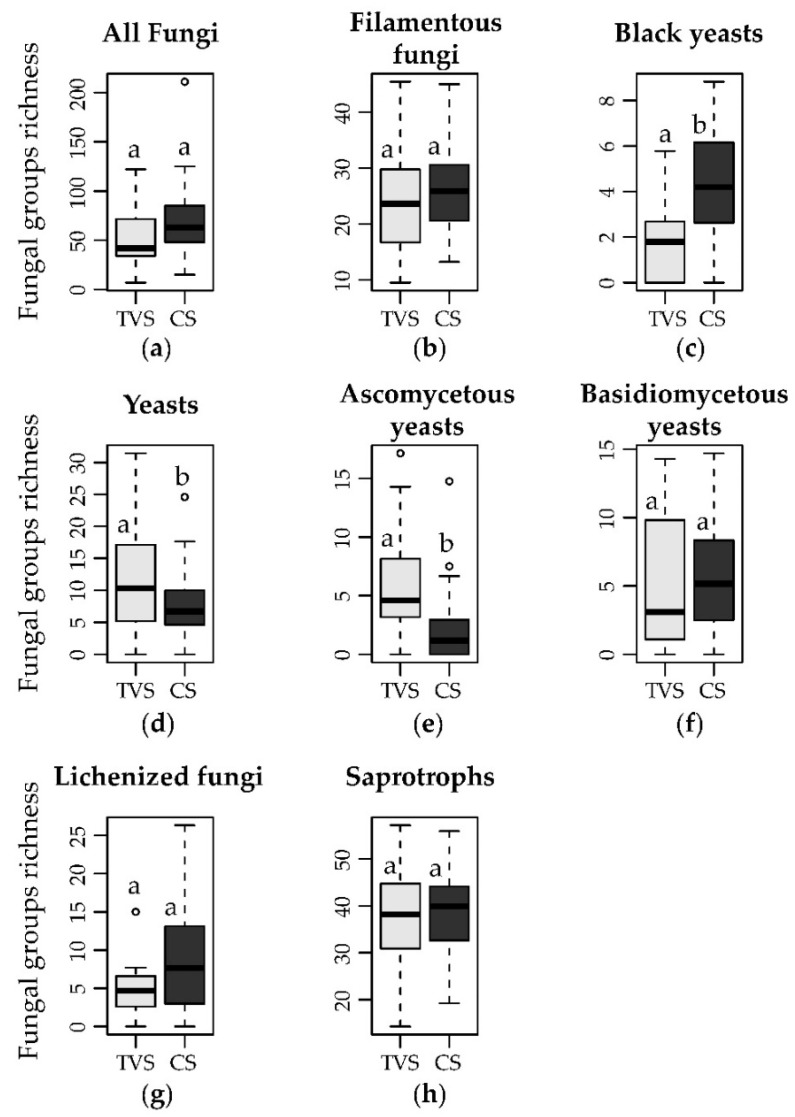
Richness differences between the two environments investigated. Richness of (**a**) the total fungal communities and relative richness (%) of (**b**–**f**) the components of the growth forms categories and (**g**,**h**) functional groups in the two different environments (TVS: Taylor Valley sites; CS: coastal sites) are reported.

The dataset obtained consisted of 860 OTUs with at least 70% identity with known fungal species hypotheses (SH). Out of 860 OTUs, 329 were present in only one of the samples and only seven in at least 50% of the samples. A total of 568 OTUs (66% of the total) were found only in samples from CS, 138 (16% of the total) only in samples from TVS, and 154 (17.9% of the total) in samples from both the environments. We found no significant (*p* < 0.05) difference in the richness between the two environments (Figure 2a), but TVS samples exhibited a significantly (*p* < 0.05) higher evenness than CS ones (Figure S1a).

**Figure 3:**
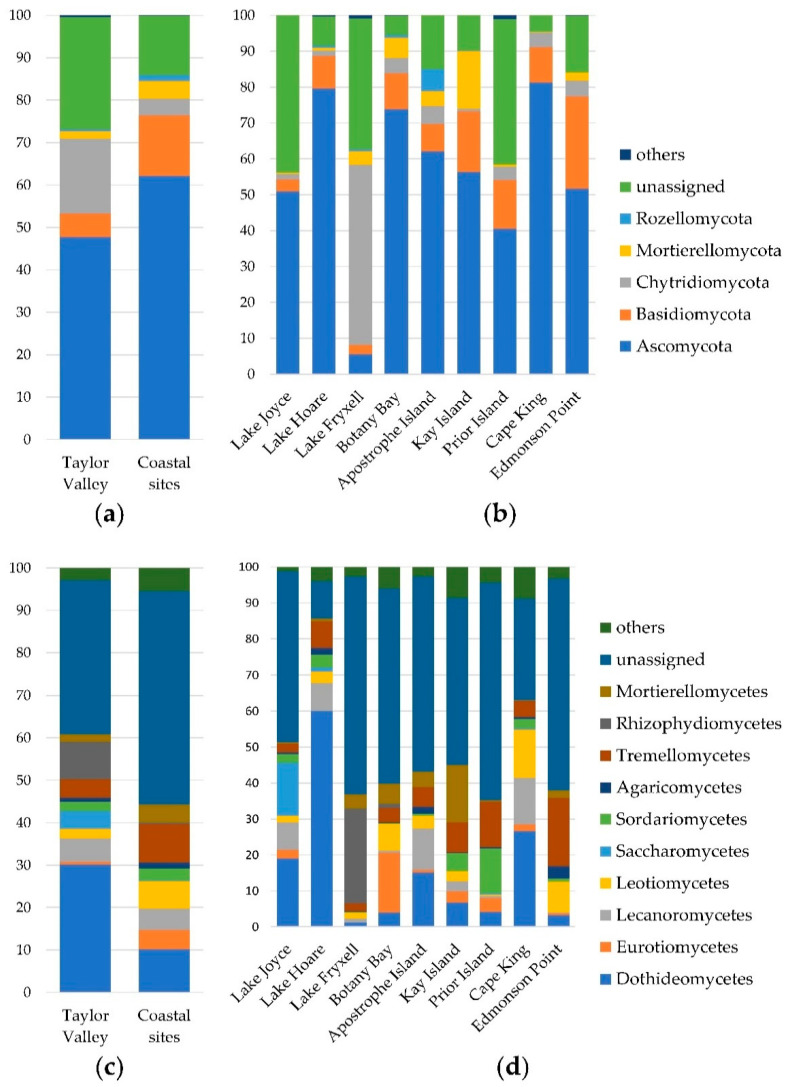
Taxonomic composition at phylum and class level. The different panels represent: (**a**) the distribution of reads at phylum level in the two different environments (Taylor Valley and coastal sites) and (**b**) in the different sampling localities, and at class level (**c**) in the two different environments (Taylor Valley and coastal sites) and (**d**) in the different sampling localities.

**Figure 4:**
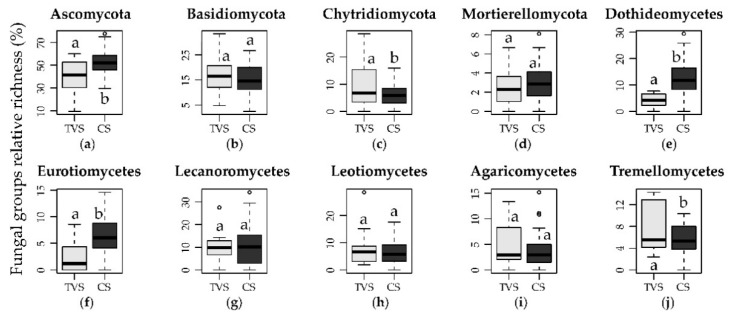
Differences in the relative richness of the most abundant fungal phyla (**a**–**d**) and classes (**e**–**j**) in the two different environments (TVS: Taylor Valley sites; CS: coastal sites).

At phylum level, 190 OTUs (22.1% of the total) matched any reference sequences (Figure 3a), representing more than 50% of all reads in eight samples out of 62. Ascomycota (418 OTUs in total) was the most abundant phylum in almost all samples, with a mean abundance of 59.28 ± 28.36% reads per sample and representing 47.7% and 62.1% of total reads for TVS and CS, respectively (Figure 3a). This phylum exhibited a significantly (*p* < 0.05) higher richness in CS compared to TVS, although the abundance showed no significant differences (Figure 4a, Figure S2a). Basidiomycota (146 OTUs) was the second most abundant phylum (12.66 ±1 2.94% of total reads per sample). It represented a mean of 5.59 and 14.35% of reads for TVS and CS, respectively (Figure 3a) and exhibited a significant (*p* < 0.05) difference of abundances between the two environments, but not of the richness (Figure 4b, Figure S2b). Other minor phyla were Chytridiomycota (6.57 ± 14.55% of total reads per sample) and Mortierellomycota (3.76 ± 10.25% of total reads per sample), represented by 57 and 20 OTUs, respectively. The former represented a mean of 17.67% and 3.90% of reads for TVS and CS, respectively, with abundances significantly different and a higher richness in TVS samples (Figure 3a and Figure 4c and Figure S2c). On the other hand, Mortierellomycota represented a mean of 1.70 and 4.25% reads for TVS and CS, respectively, but neither abundance nor richness were significantly different between the two environments (Figure 3a and Figure 4d and Figure S2d).

Ascomycota was the prevalent phylum in all different localities, ranging from 5.53% to 81.24% mean reads, followed by Basidiomycota (from 2.68% to 25.85% of reads), with the only exception being Lake Fryxell, where a prevalence of Chytridiomycota (50.11%) was observed (Figure 3b).

At class level, unassigned OTUs represented more than 50% of total reads in 33 samples out of 62 and more than 80% in seven samples. Dothideomycetes (14.7 ± 24.55%, mean abundance of total reads among all samples, 30.14% and 10.22% in TVS and CS, respectively) was the most abundant class within Ascomycota, with a great variability among the TVS samples (Figure 3c,d). Other lesser represented classes were: (i) Leotiomycetes (5.76 ± 9.62%, mean abundance of total reads among all samples, 2.23% and 6.60% in TVS and CS, respectively); (ii) Lecanoromycetes (5.06 ± 10.17%, mean abundance of total reads among all samples, 5.54 and 4.95% in TVS and CS, respectively); and (iii) Eurotiomycetes (3.76 ± 10.89%, mean abundance of total reads among all samples, 0.64 and 4.51% in TVS and CS, respectively) (Figure 3c). Non-significant (*p* > 0.05) differences were found by comparing the abundances of the different ascomycetous classes in the two different macro areas (Figure S2), while richness values of Dothideomycetes and Eurotiomycetes were significantly (*p* < 0.05) higher in CS samples (Figure 4).

Basidiomycota were mostly represented by the classes Tremellomycetes (8.28 ± 10.48% mean abundance of total reads among all samples, 4.43% and 9.20% in TVS and CS, respectively) and Agaricomycetes (1.23 ± 2.66% mean abundance of total reads among all samples, 0.88% and 1.31% in TVS and CS, respectively) (Figure 3c). Similar to Ascomycota classes, non-significant (*p* > 0.05) differences were found by comparing the abundances of the different basidiomycetous classes in the two macro areas (Figure S2), but Tremellomycetes had a significantly (*p* < 0.05) higher richness in TVS (Figure 4j). Mortierellomycetes was the most representative class within Mortierellomycota (3.76 ± 10.26% mean abundance of total reads among all samples, 1.70 and 4.25% in TVS and CS, respectively) (Figure 3c), while Rhyzophydiomycetes (Chytridiomycota) had a sporadic distribution among the samples and were almost exclusively present in Lake Fryxell in TVS (with a mean abundance of 26.38% of total reads) (Figure 3c,d). At the order level, Caliciales, Chaetothyriales, Helotiales, Mortierellales, and Pleosporales were prevalent in CS soils, while Capnodiales, Eurotiales, Hypocreales, Lecanorales, Rhizophydiales, Saccharomycetales, and Tremellales were prevalent in TVS soils (Figure S3). More than 50% of all reads could not be assigned to order level.

### 3.2. Distribution of the Different Growth Forms and Lifestyles

A total of 336 out of 860 OTUs (39.1% of the total), with at least 93% similarity to known fungal genera, were assigned to different growth forms: 241 OTUs to filamentous fungi, 64 to yeasts, and 31 to black yeasts. Among them, no significant (*p* > 0.05) differences on the richness of filamentous fungi between coastal and inland sites were found (Figure 2b), although their evenness was significantly (*p* < 0.05) higher in inner sites (Figure S1b). Instead, black yeasts showed a significantly (*p* < 0.05) higher richness in CS (Figure 2c), and an opposite trend for the evenness (Figure S1c). Yeasts exhibited a significantly (*p* < 0.05) higher richness and evenness in TVS; the richness of ascomycetous yeasts showed the same trend, while not significant (*p* > 0.05) differences were found for basidiomycetous yeasts (Figure 2d–f). The evenness of ascomycetous yeasts showed not significant (*p* > 0.05) differences between the two macro areas, while basidiomycetous yeasts followed the trend of total yeasts with a significantly (*p* < 0.05) higher evenness in TVS samples (Figure S1d–f). No significant (*p* > 0.05) difference was found on the abundance of the above-mentioned groups between the two environments (Figure S4).

A total of 461 OTUs out of 860 (54.53% of the total), with at least 90% similarity to known fungal SHs, were assigned to different lifestyles. Among them, saprotrophs and lichenized fungi were dominant, with 324 and 60 OTUs, respectively. Additionally, 16 OTUs were assigned to animal pathogens, 32 to mycorrhizal fungi, 13 to fungal parasites, and 24 to both plant pathogens and symbionts. Both saprotrophs and lichenized fungi exhibited no significant (*p* < 0.05) differences for their richness and abundance between the two environments (Figure 2g,h, Figure S4f,g), but their evenness was significantly higher in TVS samples (Figure S1g,h). On the other hand, no statistical analysis was carried out on minor functional groups (i.e., animal pathogens, mycorrhizal fungi, fungal parasites, plant pathogens, and symbionts) because of their low abundance and sporadic presence in different samples.

### 3.3. Correlation between Fungal Richness/Abundance and Edaphic Parameters

**Figure 5:**
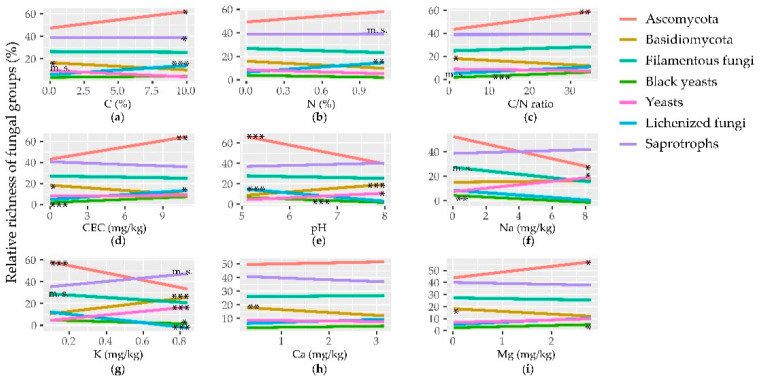
Regression lines for the variation of relative richness (*y*-axis) of the two dominant phyla, the growth forms, and the functional guilds in response to soil chemical parameters. The different panels report the correlations with: (**a**) C; (**b**) N; (**c**) C/N ratio; (**d**) cation exchange capacity (CEC); (**e**) pH; and exchangeable (**f**) Na, (**g**) K, (**h**) Ca, and (**i**) Mg (*x*-axis). The significance of the regressions is indicated as *** *p* < 0.001, ** *p* < 0.01, * *p* < 0.05, m. s. (marginally significant) *p* < 0.1. Slopes and r^2^ values of the regressions are reported in Table S1.

Edaphic parameters measured for the two macro areas are reported in Canini et al. [20,21]. Different correlations were found among the edaphic parameters and the main taxonomic groups. C content was marginally or significatively correlated with the richness of many taxonomic groups, but it was never correlated with their abundance (Figure 5 and Tables S1 and S2). N content showed correlations with the richness of some fungal classes, but only with the abundance of Tremellomycetes (Figure 5 and Tables S1 and S2). C/N ratio, which can be treated as an approximate indicator of the quality of organic matter in soils, was correlated with the richness of the two most abundant phyla with opposite trends and with few classes (with the same trends of the phyla they belong to) (Table S1). It was also positively and negatively correlated with the abundance of Ascomycota and Tremellomycetes, respectively (Figure 5 and Table S2). Cation exchange capacity (CEC) exhibited similar correlations with both the richness and abundance of the two most represented phyla, but positive with Ascomycota and negative with Basidiomycota. The CEC trends were the same for the classes of these two phyla (Figure 5 and Tables S1 and S2). pH had a marginally significant negative correlation with the total richness, and was correlated with nearly all the taxonomical groups, with opposite trends for the two major phyla and their relative classes. All the four exchangeable cations measured strongly correlated with many of the taxonomic groups tested, mainly negatively with many taxa and with K+ showing the strongest effects (Figure 5 and Tables S1 and S2). Also, the four granulometry categories showed significant (*p* < 0.05) but weak correlations with some groups, more for richness than for abundance. Finally, soil moisture had a positive marginally significant correlation with the total richness, and among taxonomical groups it was significant (*p* < 0.05) only for the abundance of Mortierellomycota (Tables S1 and S2).

Regarding the different growth forms, both richness and abundance of filamentous fungi appeared mostly uncorrelated with all parameters tested. C content was marginally negatively correlated with the richness of all yeasts and, within the group, with the ascomycetous ones. N content was correlated with neither the richness nor the abundance of the identified categories, while C/N ratio had a positive correlation with black yeasts richness. pH and the content of the four exchangeable cations correlated greater with richness than abundance of the different growth forms, with K showing the strongest effects and CEC only correlated with black yeasts richness. Finally, the four soil granulometries were mostly correlated with the richness of all yeasts and both Ascomycetous and Basidiomycetous ones (Figure 5 and Tables S3 and S4).

As for the two functional groups, abundance of saprotrophs was not correlated with any of the parameters tested. C and N contents were positively and negatively correlated with the richness of lichenized fungi and saprotrophs, respectively, while their ratio was only marginally correlated with the richness of lichenized fungi (Figure 5 and Tables S3 and S4). pH was negatively correlated with both the abundance and richness of lichenized fungi and, among the four exchangeable cations, Na+ was not correlated with the two groups, and K showed the strongest significance for the richness of both of them. Among the four soil granulometric variables, clay was not correlated with the two functional groups, while the other three categories were correlated with the richness of both groups, with opposite trends (Figure 5 and Tables S3 and S4).

### 3.4. Effect of Edaphic Parameters on Community and Groups Composition

**Figure 6:**
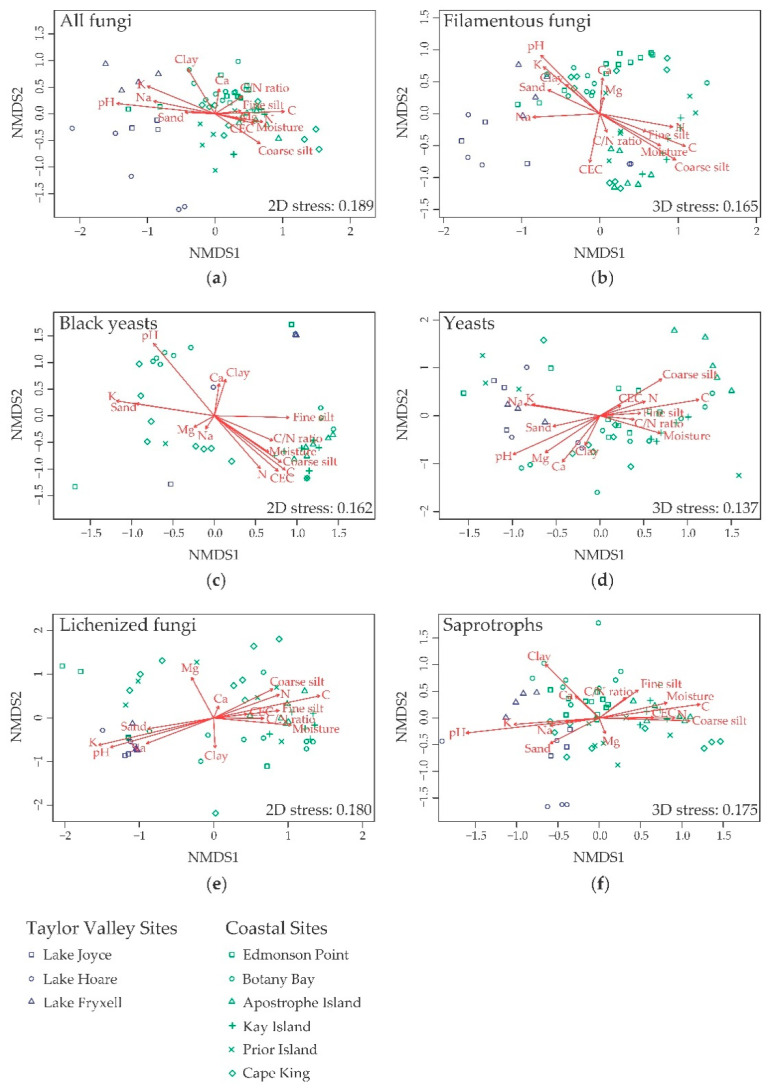
Nonmetric multidimensional scaling (NMDS) ordinations of the differences (Bray–Curtis distance) in fungal community composition (Hellinger transformed operational taxonomic units (OTUs) abundances) in the two different environments (Taylor Valley: Blue; coastal sites: Green) and different localities (different symbols). The different panels report ordinations for the total fungal community (**a**), the different growth forms categories (**b**–**d**), and functional guilds (**e**,**f**). The significance and the strength of the correlation of all the variables in the figure are reported in Table S5.

The total fungal communities, the different growth forms (filamentous fungi, yeasts, and black yeasts), and the two main lifestyles (lichenized fungi and saprotrophs) were well differentiated among the different localities, as shown in the NMDS ordinations (Figure 6). The trend was confirmed by MRPP analyses, which showed a stronger differentiation among the localities (A = 0.204 for the total fungal community, A = 0.386 for black yeasts, A = 0.252 for yeasts, A = 0.221 for filamentous fungi, A = 0.2255 for lichenized fungi, A = 0.2117 for saprotrophs; all *p* < 0.001) than between the two macro areas (A = 0.050 for the total fungal community, A = 0.089 for black yeasts, A=0.056 for yeasts, A = 0.033 for filamentous fungi, A = 0.09053 for lichenized fungi, A = 0.03558 for saprotrophs; all *p* < 0.001).

PerMANOVA analysis was carried out to estimate the proportion of variation of community composition explained by physicochemical parameters. When they were considered independently, all tested edaphic parameters showed significant (*p* < 0.05) correlations with community composition of all fungi, black yeasts, filamentous fungi, and saprotrophs. On the contrary, C/N ratio and CEC, and N, C/N ratio, CEC, and Mg were not associated with differences in the composition of lichenized fungi and yeasts, respectively (Table S6). A lower number of parameters gave significant results (*p* < 0.05) when basidiomycetous and ascomycetous yeasts were considered independently (Table S6). When the parameters were combined to account for correlations among them, pH showed the strongest correlation with total fungal community composition, the three main growth forms, and for saprotrophic fungi. Within yeasts, this was confirmed only for basidiomycetous yeasts, while for ascomycetous yeasts the result was one of the weakest independent parameters (Table 1). The strongest effect of pH was confirmed also on the two most abundant phyla (Table S7). For the total fungal community, almost all the parameters tested had an independent effect on communities variance (except for N content and C/N ratio), and all showed similar values of explained variance. A similar situation was found for filamentous and saprotrophic fungi (Table 1). For black yeasts also coarse silt, Mg and soil moisture had a not independent result from other parameters, and K, Ca, and clay explained higher proportions of variance compared to other independent variables. For yeasts, few parameters had an independent result, with soil moisture being the second strongest one (Table 1). Finally, for lichenized fungi, the effect of variables N, Na, and Ca on the observed variance had a not independent result from the effect of the other variables tested, and K content explained the highest proportion of variance (Table 1).

## 4. Discussion

Highly differentiated fungal communities characterized the two macro areas investigated. A fungal core community, composed of those OTUs occurring in at least 75% of the samples, was not recorded, either considering the totality of sites or considering those belonging to each of the two macro areas separately, suggesting that each site was characterized by its own physiognomy.

While fungal communities in coastal and inland sites showed comparable richness, pronounced compositional differences suggested strong environmental filtering determined by the contrasting environmental conditions, such as the presence/absence of biological crusts and the resulting differences in physicochemical soil parameters. The compositional difference was also evident in the difference between the numbers of OTUs exclusively found either in coastal or Taylor Valley sites (66% and 16% of the total, respectively). The combination of high richness and low evenness in fungal communities in both environments (Figure 2a, Figure S1a) suggested that only a few species have wide niche breadth and are dominant in both regions. In general, most species seemed to be highly adapted to their habitat and may have relatively low resilience to changes in environmental conditions, as previously reported for endolithic communities [44,45].

Taxonomic assemblages confirmed previous data for both continental and maritime Antarctica, with a prevalence of Ascomycota, followed by Basidiomycota, Mortierellomycota, and Chytridiomycota [45,46]. Dothideomycetes and Eurotiomycetes, the former being the most abundant class within Ascomycota in both macro areas, have also been reported as the most abundant components of Antarctic cryptoendolithic fungal communities along Victoria Land [45], as well as of permafrost collected in the McMurdo Dry Valleys and soil sediments of fumarolic ice caves on Mt. Erebus [47,48].

Many of the taxa found in this study represent classes and genera whose presence in cold (including polar) environments has already been observed. Despite that, we reported for the first time the presence in Antarctic soils of the genera *Vermiconidia*, *Friedmanniomyces,* and *Meristemomyces* (order Capnodiales), previously found in these environments only as components of endolithic communities. The genus *Vermiconidia*, present in both macro areas in seven out of nine localities, comprises saxicolous species previously found in different biomes, such as rocks in Mallorca (Spain), high mountains in the Alps, Antarctic desert, and Italian stone monuments [49,50]. The species *Friedmanniomyces endolithicus*, recorded in both the environments in five out of nine localities, is a highly melanized fungus endemic of Antarctica, previously found in endolithic communities of Victoria Land, including the McMurdo Dry Valleys [51]. The species *Meristemomyces frigidus*, occasionally present at Apostrophe and Kay Islands, was originally found in rocks from the Himalayas and later observed as a component of south-exposed Antarctic endolithic communities of inland Victoria Land [51,52]. Among the other species belonging to Dothideomycetes found in this study, *Didymella boeremae* (basionym: *Phoma boeremae*) (order Pleosporales), recorded in soils of all the coastal localities except for Prior Island, was never observed before in Antarctic sites. Finally, *Cryomyces antarcticus*, only recorded at Lake Joyce, is a black yeast-like endemic and psychrophilic fungus that deserves to be mentioned for its extraordinary ability to survive under stressful conditions, including space and Mars-simulated environments [53].

The presence and significance of the distribution of different fungal taxa in several Antarctic habitats has been the subject of some studies and speculation for many years. The dissemination in Antarctic soils of many genera previously reported in cryptoendolithic communities may be due to spreading of rock fragments due to bio-weathering processes. Antarctic rocks could be considered microbial hotspots, possibly contributing to the soil diversity. For example, Campbell and Claridge [1] reported the presence of considerable material derived from both Beacon Supergroup and dolerite in soils of Taylor, Wright, and Victoria Valleys and attributed this presence to both the weathering process and the upper atmospheric circulation. Additionally, the possibility to cover long distances by a large number of viable spores was demonstrated also for bacteria by aerobiological studies [54,55]. The propagules’ survival should be greater when both fungi and bacteria are dispersed within rock fragments.

Lecanoromycetes and Leotiomycetes, comprised of some lichenized fungi, were abundant taxa within Ascomycota and were found in all the samples analyzed with no statistical differences for their abundance and richness between the two macro areas. Unlike Leotiomycetes, Lecanoromycetes were frequently recorded in Antarctic soils. Lichenized fungi are important components of Antarctic soils [24] and Lecanoromycetes is the most diverse lichen-forming class in Antarctica. This class is also widely distributed in soil samples of the Prince Charles Mountains in the southern part of Mac Robertson Land, East Antarctica [24]. The McMurdo Dry Valleys and Queen Maud Mountains were suggested as possible glacial refugia for Lecanoromycetes due to a combination of prevailing wind patterns and physical barriers restricting spore dispersal, recruitment, and settlement [56]. Being a common inhabitant of rocks, they might be present in soils as the result of rock fragment dissemination due to bio-weathering processes, as hypothesized before for Dothideomycetes, or of air dispersed lichen thalli fragments. In this framework, it was hypothesized that the “lichenosphere” (defined as the non-lichenized fungal communities associated with the Antarctic lichens) could be a protected natural niche representing, again, a microbial hotspot for survival and air dispersion of microorganisms in the Antarctic environments [57].

Both Tremellomycetes and Agaricomycetes (phylum Basidiomycota) were already found in rock samples in Northern Victoria Land [45], where Tremellomycetes had a relative abundance value (10%) close to that recorded for CS here (9.2%). Tremellomycetes encompasses a number of yeast genera exhibiting either psychrophilic or psychrotolerant aptitude, namely *Mrakia*, *Naganishia*, *Solycoccozyma*, and *Dioszegia*, including many species occurring in the Antarctic continent [32,58,59,60].

Regarding the different growth forms, filamentous fungi dominated over yeasts and black yeasts, both in terms of richness and abundance in both macro areas. They probably included, among others, many cosmopolitan species, air transported as propagules, some of which are probably unable to grow under Antarctic conditions. Interesting to note was the significantly (*p* < 0.05) greater richness of yeasts in TVS than CS. This evidence was possibly due to the more stressful conditions of the former sites. Despite the fact that basidiomycetous yeasts are known to be dominant in polar soils, being more tolerant to low temperatures [61,62], the greater richness in TVS appeared to be determined mainly by ascomycetous yeasts (Figure 2e), which are generally considered more xerotolerant [63]. Culture-dependent studies reported that their abundance and diversity is related to water activity [62]. Therefore, lower moisture contents, as in Taylor Valley soils, could give rise to the observed greater diversity of ascomycetous yeasts in these sites. Moisture did not appear to be an independent parameter in determining ascomycetous yeast distribution as these fungi are strongly affected by soil granulometry, which, in turn, influences water retention, explaining the observed trend. The highest evenness, which was recorded for Ascomycetous yeasts in both environments, could suggest their high resilience to environmental changes.

In general, higher evenness characterized both growth forms and lifestyles groups compared to the total fungal communities, with almost always significantly higher values for TVS. Additionally, all the main taxonomic groups showed a greater ability to react to changes of the stressful environment of inner sites compared to coastal sites (Figure S1). This raises several questions on the possible effects of climate change on the stability of the soil biological crusts of the coastal sites versus the inland sites, and it is in disagreement with the idea that increasing complexity in a system leads to an increased robustness of the community to disturbance [64]. This result should be investigated further, ideally using different approaches distinguishing fungi that are active in soils from those that are relics or present in dormant stages.

Black yeasts have morphological and physiological characteristics, making them among the organisms best adapted to the harsh Antarctic conditions [35]. The significant difference recorded in their richness between the two regions reflected the differences in the richness values of the two classes, Dothideomycetes and Eurotiomycetes, in which most of them are distributed. They have been commonly recorded within cryptoendolithic communities, which dominate the areas where the environmental conditions become incompatible with surface colonization [15]. Therefore, their growth seemed to be inhibited in soil, possibly due to its unconsolidated nature compared to rocks, where their presence might be restricted to filamentous growth. In fact, they are characterized by a wide environmental plasticity and may promptly shift from one growth form to another according to the physicochemical environmental conditions as an overlooked adaptive strategy to stressful conditions [65,66]. Their lower abundance compared to filamentous and yeasts fungi is compatible with their lower growth rates. The high evenness observed for black yeasts in Taylor Valley sites suggested that most of the low number of species were common throughout the region, and, therefore, they appeared to tolerate greater differences in environmental conditions in the inland than in coastal sites. This may be due to the more favorable environment for life in general of coastal sites, where there may be more intense competition for resources among black yeasts and other microorganisms, and these competitive interactions may be influenced by local variations in environmental conditions.

Regarding the different lifestyles, saprotrophic and lichenized fungi were the two main groups in both regions, which was in agreement with previous studies reporting them as widespread in Antarctica [20,21,67,68]. We found a close relationship in richness, abundance, and composition of both saprotrophic and lichenized fungi with soil pH and C and K contents. The high abundance of saprotrophs was not unexpected, due to their pivotal role as decomposers of organic matter, while the higher abundance of lichenized fungi in CS than TVS is consistent with the high abundance of lichens in BSCs of coastal sites.

Moreover, the composition at the level of the different growth forms and lifestyles were well differentiated among the two macro areas, presumably determined by the presence/absence of biological crust coverage and, in turn, the physicochemical soil parameters, as hypothesized for the total community. Considering the effect of different parameters on the observed variance of the community, pH, K, and C contents were among the parameters explaining the highest proportion of variance in community composition, with pH and K content playing a prevalent effect in determining the composition of TVS samples. C content was mainly related to the composition of coastal communities, possibly due to higher C turnover rates connected to the above crust coverage. This confirmed previous reports correlating this parameter with the abundance of culturable fungi in Antarctic soils and the nutritional limitation as the main factor influencing the distribution and abundance of native fungi in oligotrophic environments [69,70].

The variation in taxonomic assemblage of fungal communities at the level of the total community, the growth forms, and the functional guilds was also explained by other soil physicochemical parameters. Among these, soil granulometry, strongly influencing nutrient availability in such limiting conditions, showed a prominent effect. Preferences for different sized soil particles (clay, silt, and sand) have already been reported for different bacterial phyla, strongly contributing to the spatial heterogeneity and bacterial diversity found [71,72]. In fact, the almost always greater percentage of sand in all samples results in a lower ability to retain nutrient, cations, and water, and even small changes in pH or soil water content can affect the community composition. The relevant effect of abiotic parameters, widely reported for Antarctica, was already highlighted when the two regions were investigated independently [20,21].

## 5. Conclusions

To define the main parameters driving microbial community composition and dynamics in Antarctica is one of the main goals of microbiologists, even in the face of global change dramatically affecting polar environments [73]. In this work, soil fungal communities of two different regions with different climates, representative of relatively humid coastal and dry inland sites, are shown to be highly differentiated, mainly connected to differences in local abiotic parameters.

In the scope of climate change threatening to cause a loss of dry-adapted endemic oligotrophic taxa and a greater prevalence of generalist taxa, these results are of particular interest. In fact, some groups had a result of very low evenness, often associated with a very low resilience ability. Artificial increases of water and organic matter in arid soils of the McMurdo Dry Valleys, simulating expected consequences of climate changes, were demonstrated to induce changes in both bacterial and fungal components [74]. This should be taken into account given the high differentiation in the community composition of the two macro areas, since a higher water availability could result in a higher diffusion of vegetal species.

## Figures and Tables

**Table 1:** Proportion of variation in fungal community composition at the level of the total community (all fungi), the growth form categories, and functional guilds explained by soil physicochemical parameters added sequentially (first to the last) in a model, depending on their independent influence in the variance, as reported in Table S6. Par: soil parameters; V: variance; P: *p* value; R: residuals; Cs: Coarse silt; Fs: Fine silt; Moist: moisture.

All FUNGI			Black YEASTS			Filamentous FUNGI			All YEASTS			Ascomycetous YEASTS			Basidiomycetous YEASTS			Lichenized FUNGI			Saprotrophs		
Par	V	*p*	Par	V	*p*	Par	V	*p*	Par	V	*p*	Par	V	*p*	Par	V	*p*	Par	V	*p*	Par	V	*p*
pH	7.93	0.0001	pH	18.9	0.0001	pH	9.676	0.0001	pH	8.23	0.0001	Clay	23.186	0.0001	pH	7.91	0.0001	K	13.239	0.0001	pH	8.774	0.0001
C	3.555	0.0002	C	4.678	0.0002	C	4.621	0.0001	Moist	6.038	0.0002	Fs	4.75	0.0301	K	3.904	0.017	pH	5.534	0.0004	C	3.459	0.0002
Cs	4.284	0.0001	K	7.263	0.0001	Cs	4.264	0.0001	K	3.774	0.0052	Sand	4.126	0.0562	Moist	5.672	0.0007	C	5.293	0.0002	K	3.946	0.0001
K	3.687	0.0001	Cs	1.493	0.1663	K	3.851	0.0001	Sand	5.532	0.0005	Ca	1.578	0.413	C/N	2.838	0.0802	Cs	2.551	0.0319	Cs	3.653	0.0002
Na	3.259	0.0003	N	1.784	0.0858	Moist	3.073	0.0002	Fs	3.676	0.0093	Moist	1.803	0.3874	Na	2.563	0.1102	Moist	3.182	0.0072	CEC	4.256	0.0001
Moist	2.815	0.0009	Clay	7.541	0.0001	CEC	4.744	0.0001	C	0.872	0.7834	K	3.412	0.1036	Ca	4.781	0.0045	Na	2.003	0.0651	Clay	5.097	0.0001
Sand	3.957	0.0002	CEC	3.173	0.0051	Ca	4.323	0.0001	Cs	1.713	0.2634	Na	7.937	0.0013	Fs	3.004	0.0653	Mg	6.968	0.0001	Moist	2.881	0.0004
Clay	3.103	0.0004	Ca	6.085	0.0002	N	1.91	0.0306	Na	4.142	0.0015	pH	4.516	0.0404	R	69.329		N	1.798	0.1495	Ca	2.534	0.002
Fs	5.76	0.0001	Sand	4.523	0.0002	Mg	5.374	0.0001	Clay	3.752	0.0071	R	48.692					Sand	4.858	0.0005	Na	3.337	0.0001
N	1.482	0.1115	Fs	4.343	0.0005	Sand	2.333	0.0061	Ca	4.092	0.0048							Fs	3.287	0.0066	N	1.625	0.064
CEC	4.21	0.0001	Na	2.151	0.0329	Clay	4.677	0.0001	R	58.179								Ca	0.714	0.8647	Mg	4.757	0.0001
Ca	4.314	0.0001	Moist	2.042	0.0587	Fs	2.06	0.0106										Clay	2.669	0.0271	Sand	5.489	0.0001
Mg	2.797	0.0005	Mg	0.739	0.6523	C/N	1.566	0.1026										R	47.905		Fs	2.58	0.0009
C/N	1.572	0.0805	C/N	0.83	0.5776	R	47.53														C/N	1.35	0.1885
R	47.312		R	34.454																	R	46.261	

## Data Availability

The data presented in this study are openly available in [NCBI gene bank] reference numbers [MK536607–MK537296 and MT586999–MT587274].

## Supplementary Materials

The following are available online at https://www.mdpi.com/article/10.3390/biology10040320/s1, Figure S1: Evenness of the total fungal communities and of the components of the growth forms categories and functional groups in the two different environments, Figure S2: Relative abundance (%) of the most abundant fungal phyla and classes in the two different environments, Figure S3: Distribution of identified OTUs at order level in the two different environments (a) and in the different sampling localities (b), Figure S4: Relative abundance (%) of the components of the growth forms categories and of the functional groups in the two different environments, Table S1: Regression slopes for the variation of relative richness of the total fungal community and relative richness of most abundant phyla and classes in response to edaphic parameters, Table S2: Regression slopes for the variation of relative abundance of most abundant phyla and classes in response to edaphic parameters, Table S3: Regression slopes for the variation of relative richness of the growth forms categories and the functional guilds in response to edaphic parameters, Table S4: Regression slopes for the variation of relative abundance of the growth forms categories and the functional guilds in response to edaphic parameters, Table S5: Regression r2 and significance of each variable fitted in the NMDS ordinations, Table S6: Proportion of variation in fungal community composition, at the level of the total community (all fungi), the growth form categories, and the functional guilds explained by soil physicochemical parameters calculated independently, Figure S7: Proportion of variation in fungal community composition, at the level of the most abundant phyla explained by soil physicochemical parameters added sequentially (first to the last) in a model depending on their independent influence in the variance, Figure S8: Proportion of variation in fungal community composition, at the level of the most abundant phyla explained by soil physicochemical parameters calculated independently.
