# Supplementary material for: Growth Forms and Functional Guilds Distribution of Soil Fungi in Coastal Versus Inland Sites of Victoria Land, Antarctica
Source: Biology (Basel). 2021 Apr 11;10(4):320. doi: 10.3390/biology10040320 (PMC8070035; doi:10.3390/biology10040320)
Supplement: Supplementary file 1 [file biology-10-00320-s001.pdf]

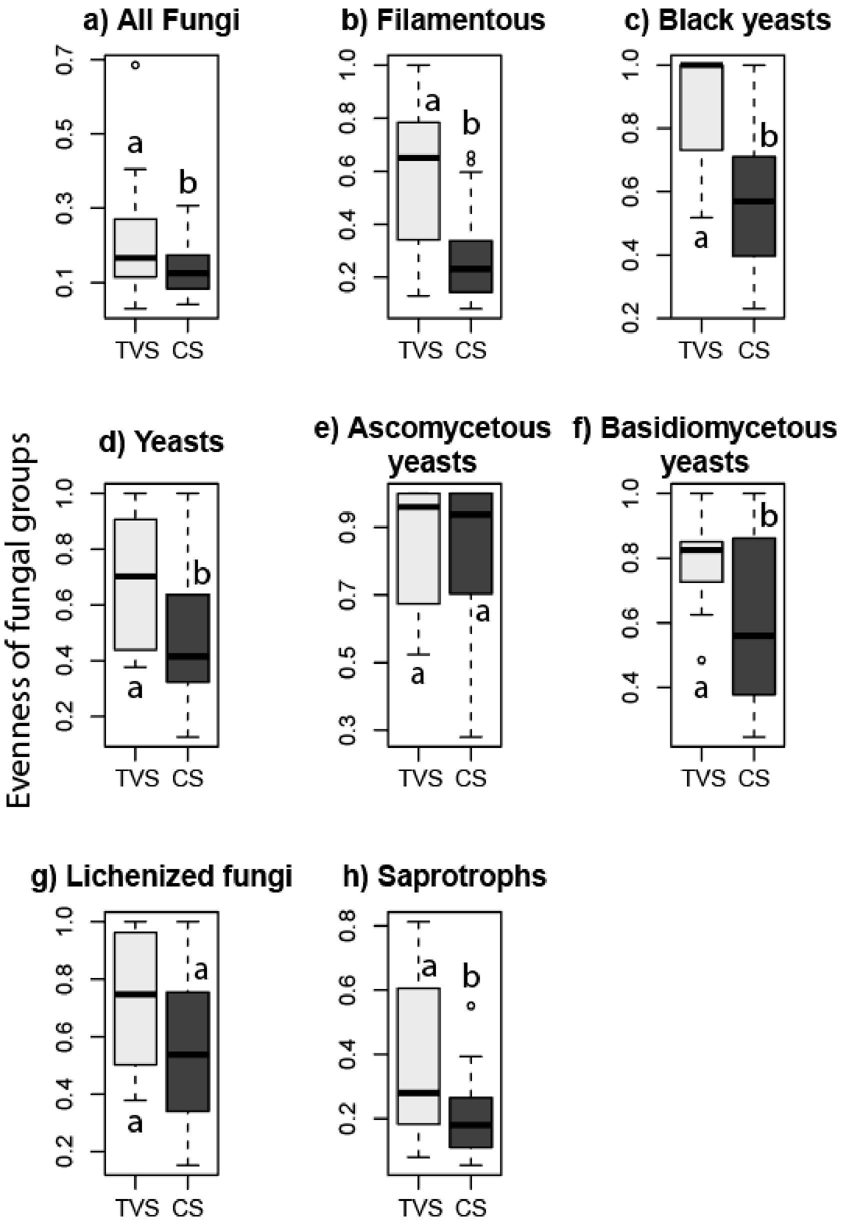

Figure S1. Evenness of the total fungal communities and of the components of the growth forms categories and functional groups in the two different environments (TVS: Taylor Valley Sites; CS: Coastal Sites).

Supplementary material

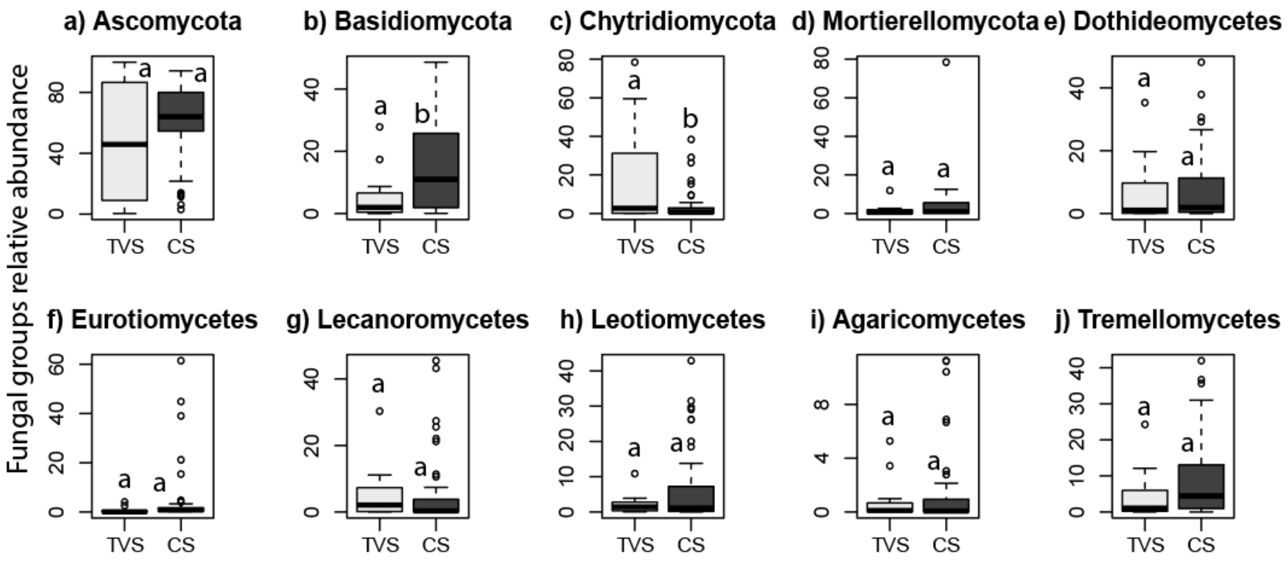

Figure S2. Relative abundance (%) of the most abundant fungal phyla and classes in the two different environments (TVS: Taylor Valley Sites; CS: Coastal Sites).

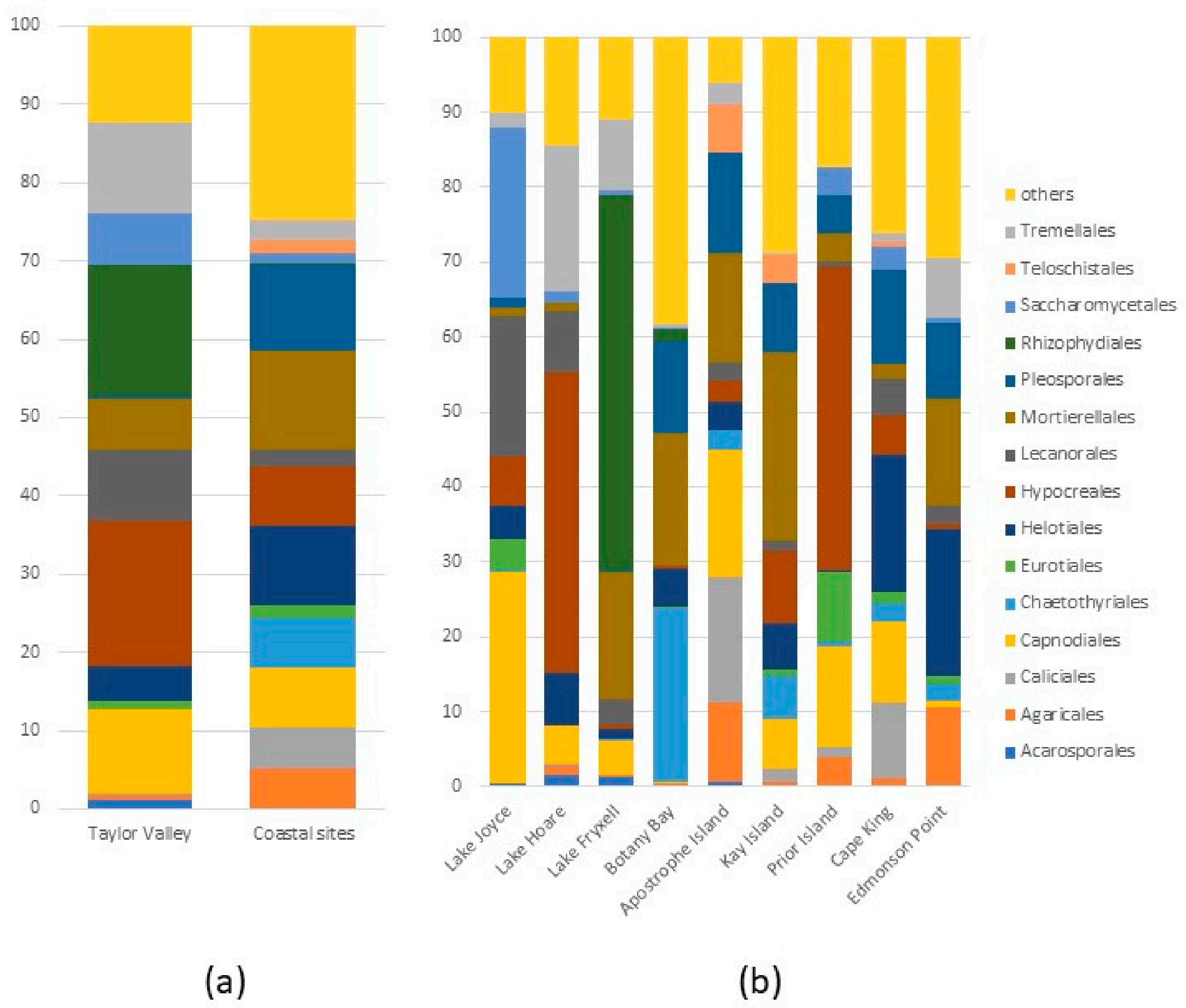

Figure S3. Distribution of identified OTUs at order level in the two different environments (a) and in the different sampling localities (b).

Supplementary material

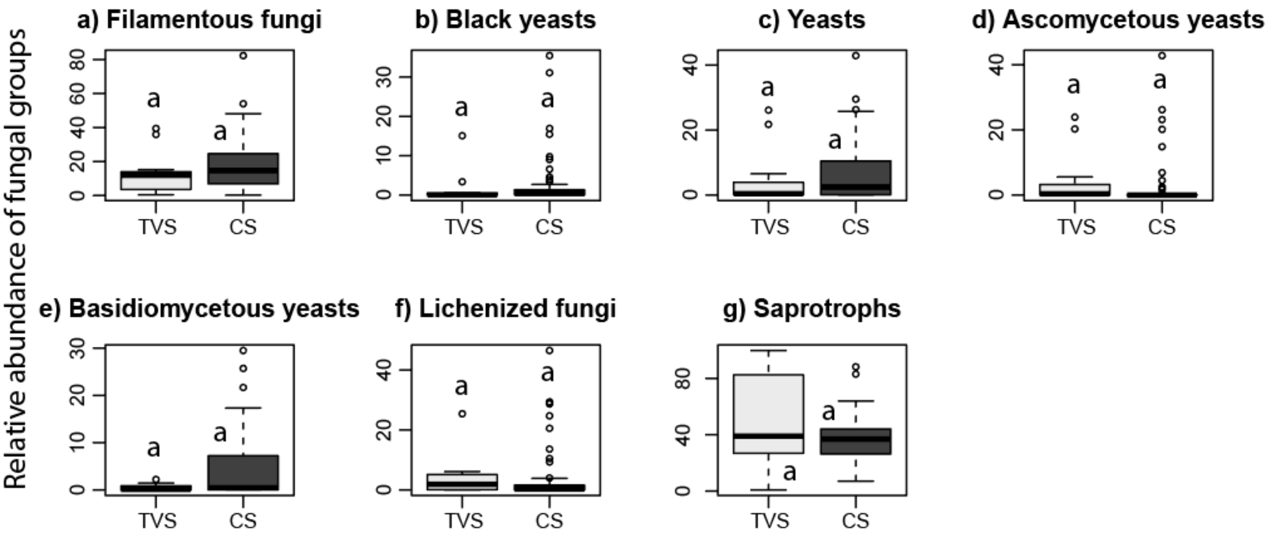

Figure S4. Relative abundance (%) of the components of the growth forms categories and of the functional groups in the two different environments (TVS: Taylor Valley Sites; CS: Coastal Sites).

Supplementary material

Table S1. Regression slopes for the variation of relative richness of the total fungal community and relative richness of most abundant phyla and classes in response to edaphic parameters (significant for $p < 0.05$).

Soil parameters		All fungi	Ascomycota	Basidiomycota	Chytridiomycota	Mortierellomycota	Dothideomycetes	Eurotiomycetes	Lecanoromycetes	Letiomycetes	Agaricomycetes	Tremellomycetes
C	slope<br>r^2	n. s.	1.4617<br>0.08462	-0.6441<br>0.05008	-0.4781§<br>0.04082	n. s.	0.7580<br>0.0656	n. s.	0.6762§<br>0.03145	-0.4574§<br>0.04291	-0.4236<br>0.09198	n. s.
N	slope<br>r^2	n. s.	n. s.	n. s.	n. s.	n. s.	7.379§<br>0.03752	n. s.	n. s.	-6.4292<br>0.06592	-3.6144§<br>0.03819	n. s.
C/N ratio	slope<br>r^2	n. s.	0.4555<br>0.12	-0.18819<br>0.06249	n. s.	n. s.	n. s.	0.17281<br>0.1942	0.1968§<br>0.04001	n. s.	-0.11070<br>0.08618	n. s.
CEC	slope<br>r^2	n. s.	1.952<br>0.1571	-0.8275<br>0.08945	n. s.	n. s.	n. s.	0.5974<br>0.1574	1.1468<br>0.1175	n. s.	-0.4897<br>0.1228	n. s.
pH	slope<br>r^2	-9.659§<br>0.03956	-9.180<br>0.3488	3.6501<br>0.1799	2.5106<br>0.1288	n. s.	-3.985<br>0.1914	-1.9601<br>0.1611	-4.386<br>0.1698	1.9169<br>0.07899	1.7846<br>0.1592	n. s.
Na	slope<br>r^2	n. s.	-3.044<br>0.08884	n. s.	n. s.	n. s.	-1.5306<br>0.06386	n. s.	n. s.	n. s.	n. s.	n. s.
K	slope<br>r^2	n. s.	-33.308<br>0.2046	20.087<br>0.258	n. s.	n. s.	-15.978<br>0.1372	-6.047<br>0.06077	-19.544<br>0.1539	9.595<br>0.09389	6.4411<br>0.08856	6.7191<br>0.1213
Ca	slope<br>r^2	n. s.	n. s.	-2.0694<br>0.1015	1.5641<br>0.08903	-0.5684<br>0.08001	n. s.	1.4467<br>0.165	n. s.	-1.5805<br>0.1056	-0.9298<br>0.0725	-1.190<br>0.1599
Mg	slope<br>r^2	n. s.	4.756<br>0.09109	-2.217<br>0.06293	n. s.	-1.0777<br>0.1889	2.466<br>0.07084	1.9290<br>0.1736	n. s.	-1.7134<br>0.06766	-1.4735<br>0.1156	-1.1564<br>0.08105
Moisture	slope<br>r^2	1.5176§<br>0.03548	n. s.	n. s.	n. s.	n. s.	n. s.	n. s.	n. s.	n. s.	n. s.	n. s.
Sand	slope<br>r^2	n. s.	n. s.	0.18527<br>0.1493	n. s.	n. s.	n. s.	n. s.	n. s.	0.16334<br>0.2123	0.07875<br>0.09532	0.04670§<br>0.03018
Coarse silt	slope<br>r^2	n. s.	n. s.	-0.4188<br>0.1155	-0.2607<br>0.06325	n. s.	n. s.	n. s.	n. s.	-0.3169<br>0.1176	-0.16231<br>0.05733	n. s.
Fine Silt	slope<br>r^2	n. s.	n. s.	-0.30811<br>0.1474	n. s.	n. s.	n. s.	0.10159§<br>0.03871	n. s.	-0.27333<br>0.2126	-0.12779<br>0.08873	-0.08536§<br>0.03946
Clay	slope<br>r^2	n. s.	n. s.	n. s.	0.7888<br>0.1167	n. s.	-0.7556<br>0.05226	0.6632<br>0.1725	n. s.	-0.5632<br>0.0599	n. s.	n. s.

§marginally significant.

Supplementary material

Table 2. Regression slopes for the variation of relative abundance of most abundant phyla and classes in response to edaphic parameters (significant for $p < 0.05$).

Soil parameters		Ascomycota	Basidiomycota	Chytridiomycota	Mortierellomycota	Dothideomycetes	Eurotiomycetes	Lecanoromycetes	Letiomycetes	Agaricomycetes	Tremellomycetes
C	slope	n. s.	n. s.	n. s.	n. s.	n. s.	n. s.	n. s.	n. s.	n. s.	n. s.
	r^2										
N	slope	n. s.	n. s.	n. s.	n. s.	n. s.	n. s.	n. s.	n. s.	n. s.	12.704
	r^2										0.05343
C/N ratio	slope	0.7825	n. s.	n. s.	n. s.	n. s.	n. s.	n. s.	n. s.	n. s.	-0.3065
	r^2	0.05689									0.06441
CEC	slope	2.683§	-1.5504	n. s.	n. s.	1.9183	n. s.	1.3034	n. s.	n. s.	-1.5433
	r^2	0.04294	0.07843			0.176		0.09921			0.1261
pH	slope	-10.388	n. s.	5.832	n. s.	-6.735	n. s.	-3.027§	n. s.	n. s.	n. s.
	r^2	0.06848		0.08532		0.2086		0.04218			
Na	slope	-9.066	n. s.	2.880§	n. s.	n. s.	n. s.	n. s.	n. s.	n. s.	n. s.
	r^2	0.1555		0.04871							
K	slope	-57.545	17.197§	n. s.	n. s.	n. s.	n. s.	n. s.	n. s.	n. s.	18.209
	r^2	0.1041	0.03405								0.06986
Ca	slope	n. s.	-2.818§	n. s.	n. s.	n. s.	3.479	n. s.	n. s.	-0.8581	-2.575
	r^2		0.03889				0.1016			0.1049	0.05367
Mg	slope	n. s.	-3.671§	n. s.	n. s.	n. s.	n. s.	n. s.	2.927§	-1.3818	-3.547
	r^2		0.03884						0.04702	0.1693	0.06199
Moisture	slope	n. s.	n. s.	n. s.	0.7119	n. s.	n. s.	n. s.	n. s.	n. s.	n. s.
	r^2				0.09525						
Sand	slope	n. s.	n. s.	n. s.	n. s.	n. s.	-0.17879§	n. s.	n. s.	0.047	n. s.
	r^2						0.0374			0.0466	
Coarse silt	slope	n. s.	n. s.	n. s.	n. s.	n. s.	n. s.	n. s.	n. s.	n. s.	n. s.
	r^2										
Fine Silt	slope	n. s.	n. s.	n. s.	n. s.	n. s.	0.3573	n. s.	n. s.	-0.07998§	n. s.
	r^2						0.0609			0.04888	
Clay	slope	n. s.	-1.2926§	n. s.	n. s.	n. s.	2.0289	n. s.	n. s.	-0.3359	-1.1041§
	r^2		0.04125				0.183			0.07532	0.04727

Supplementary material

§marginally significant.

Table 3. Regression slopes for the variation of relative richness of the growth forms categories and the functional guilds in response to edaphic parameters (significant for $p < 0.05$).

Soil parameters		Filamentous fungi	Black yeasts	All yeasts	Ascomycetous yeasts	Basidiomycetous yeasts	Lichenized fungi	Saprotrophs
C	slope	n. s.	n. s.	-0.5519§	-0.3697	n. s.	1.0259	-0.8898
	r2			0.03912	0.05382		0.25	0.05009
N	slope	n. s.	n. s.	n. s.	n. s.	n. s.	9.2792	-8.937§
	r2						0.1354	0.03013
C/N ratio	slope	n. s.	0.1320	n. s.	n. s.	n. s.	0.12123§	n. s.
	r2		0.2482				0.03425	
CEC	slope	n. s.	0.5037	n. s.	n. s.	n. s.	0.6683	n. s.
	r2		0.2502				0.09128	
pH	slope	n. s.	-1.7346	2.1553	1.1687§	n. s.	-3.8137	n. s.
	r2		0.2836	0.06102	0.04729		0.3185	
Na	slope	-1.4392§	-0.7109	1.4155	0.8010	n. s.	n. s.	n. s.
	r2	0.04686	0.1161	0.07196	0.0629			
K	slope	-10.932§	-4.5258	16.439	5.635	10.803	-16.969	13.486
	r2	0.04728	0.07691	0.193	0.05192	0.1915	0.289	0.04753
Ca	slope	n. s.	n. s.	n. s.	n. s.	n. s.	n. s.	n. s.
	r2							
Mg	slope	n. s.	0.9499	n. s.	1.1200§	n. s.	n. s.	n. s.
	r2		0.08224		0.04824			
Moisture	slope	n. s.	n. s.	n. s.	-0.19718	n. s.	0.2525§	n. s.
	r2				0.05189		0.03787	
Sand	slope	n. s.	n. s.	0.1425	n. s.	0.09986	-0.16325	0.1569§
	r2			0.09509		0.1096	0.1844	0.04516
Coarse silt	slope	n. s.	n. s.	-0.4295	-0.18015	-0.24936	0.4531	-0.4129
	r2			0.1422	0.06134	0.1062	0.2253	0.05026
Fine Silt	slope	n. s.	n. s.	-0.2269	n. s.	-0.16739	0.25719	-0.2426§
	r2			0.08453		0.1102	0.1617	0.03605
Clay	slope	n. s.	n. s.	n. s.	n. s.	n. s.	n. s.	n. s.
	r2							

§marginally significant.

Supplementary material

Table 4. Regression slopes for the variation of relative abundance of the growth forms categories and the functional guilds in response to edaphic parameters (significant for $p < 0.05$).

**Soil parameters**		**Filamentous Fungi**	**Black yeasts**	**All yeasts**	**Ascomycetous yeasts**	**Basidiomycetous yeasts**	**Lichenized fungi**	**Saprotrophs**
C	slope	n. s.	n. s.	n. s.	n. s.	n. s.	n. s.	n. s.
	r^2							
N	slope	n. s.	n. s.	n. s.	n. s.	n. s.	n. s.	n. s.
	r^2							
C/N ratio	slope	n. s.	n. s.	n. s.	n. s.	n. s.	n. s.	n. s.
	r^2							
CEC	slope	n. s.	n. s.	n. s.	n. s.	n. s.	n. s.	n. s.
	r^2							
pH	slope	n. s.	n. s.	n. s.	n. s.	n. s.	-1.5341§	n. s.
	r^2						0.03078	
Na	slope	n. s.	n. s.	n. s.	n. s.	n. s.	n. s.	n. s.
	r^2							
K	slope	n. s.	n. s.	n. s.	n. s.	8.9212	n. s.	n. s.
	r^2					0.03634		
Ca	slope	n. s.	1.5347§	1.953§	2.4183	n. s.	-1.6280	n. s.
	r^2		0.04271	0.03065	0.08331		0.08457	
Mg	slope	n. s.	n. s.	3.936	3.184	n. s.	-1.5578§	n. s.
	r^2			0.09776	0.08538		0.0375	
Moisture	slope	n. s.	n. s.	n. s.	n. s.	n. s.	0.3167	n. s.
	r^2						0.05992	
Sand	slope	n. s.	n. s.	n. s.	n. s.	n. s.	n. s.	n. s.
	r^2							
Coarse silt	slope	n. s.	n. s.	n. s.	-0.3633§	n. s.	n. s.	n. s.
	r^2				0.04456			
Fine Silt	slope	n. s.	n. s.	n. s.	n. s.	n. s.	n. s.	n. s.
	r^2							
Clay	slope	n. s.	0.9516	1.3358	1.44403	n. s.	n. s.	n. s.
	r^2		0.09717	0.09415	0.1605			

§marginally significant.

Supplementary material

Table 5. Regression r^2 and significance of each variable fitted in the NMDS ordinations (Figure 6).

Soil parameters	All fungi		Filamentous fungi		Black yeasts		All yeasts		Lichenized fungi		Saprotrophs	
	r^2	*p*	r^2	*p*	r^2	*p*	r^2	*p*	r^2	*p*	r^2	*p*
C	0.1838	0.008	0.3619	0.001	0.3084	0.001	0.2061	0.003	0.3997	0.001	0.2688	0.001
N	0.0910	0.069	0.2273	0.002	0.2239	0.004	0.0533	0.281	0.1848	0.005	0.1448	0.013
C/N ratio	0.0471	0.234	0.0232	0.508	0.1248	0.047	0.0237	0.542	0.0791	0.134	0.0449	0.276
CEC	0.0394	0.320	0.1503	0.009	0.2904	0.001	0.0151	0.703	0.0366	0.417	0.0751	0.103
pH	0.3719	0.001	0.3516	0.001	0.4145	0.001	0.2424	0.001	0.4120	0.001	0.4639	0.001
Na	0.1524	0.016	0.1836	0.003	0.0121	0.740	0.1193	0.049	0.2009	0.002	0.0649	0.145
K	0.2201	0.001	0.2665	0.001	0.2574	0.002	0.0963	0.078	0.4812	0.001	0.1882	0.004
Ca	0.0386	0.337	0.0797	0.102	0.0641	0.235	0.1639	0.015	0.0142	0.715	0.0450	0.281
Mg	0.0223	0.546	0.0176	0.611	0.0183	0.652	0.1447	0.024	0.1634	0.018	0.0150	0.665
Moisture	0.0625	0.168	0.2130	0.001	0.1573	0.021	0.0905	0.093	0.1937	0.006	0.1308	0.027
Sand	0.0339	0.378	0.1456	0.019	0.1645	0.019	0.0498	0.265	0.1462	0.019	0.1006	0.048
Coarse silt	0.1321	0.015	0.3642	0.001	0.2469	0.001	0.1613	0.015	0.1866	0.007	0.2085	0.004
Fine silt	0.0261	0.476	0.1073	0.039	0.1425	0.033	0.0324	0.446	0.1400	0.022	0.0869	0.071
Clay	0.1520	0.019	0.0998	0.053	0.0844	0.138	0.0587	0.235	0.0821	0.117	0.2526	0.001

Supplementary material

Table 6. Proportion of variation in fungal community composition, at the level of the total community (all fungi), the growth form categories, and the functional guilds explained by soil physicochemical parameters calculated independently. Variance was calculated with permutational multivariate analysis of variance, based on Hellinger-transformed fungal community matrix. Significant variables were included in the final model for each fungal group (Table 1).

**Soil parameters**	**All fungi**		**Black yeasts**		**Filamentous fungi**		**All yeasts**		**Ascomycetous yeasts**		**Basidiomycetous yeasts**		**Lichenized fungi**		**Saprotrophs**	
	variance	p	variance	p	variance	p	variance	p	variance	p	variance	p	variance	p	variance	p
C	**6,882046**	**0,001**	**16,16643**	**0,001**	**8,43079**	**0,001**	**5,39913**	**0,006**	3,161653	0,319	3,540147	0,082	**12,68779**	**0,001**	**6,826018**	**0,001**
N	**4,264757**	**0,001**	**11,05689**	**0,001**	**4,727565**	**0,001**	2,987655	0,11	3,926552	0,204	1,398112	0,74	**6,729929**	**0,001**	**3,941239**	**0,004**
C/N ratio	**2,832519**	**0,039**	**6,859914**	**0,003**	**3,322697**	**0,014**	3,733582	0,054	3,090943	0,305	**5,535204**	**0,008**	2,96505	0,111	**3,164755**	**0,021**
CEC	**4,237893**	**0,001**	**9,427302**	**0,001**	**5,946512**	**0,001**	3,095464	0,113	3,56126	0,288	3,490264	0,082	2,913655	0,109	**5,385856**	**0,001**
pH	**7,892664**	**0,001**	**18,9004**	**0,001**	**9,675734**	**0,001**	**8,22956**	**0,002**	**7,082999**	**0,032**	**7,909569**	**0,001**	**12,82275**	**0,001**	**8,773687**	**0,001**
Na	**4,966609**	**0,001**	**8,518388**	**0,001**	**4,325597**	**0,001**	**4,432121**	**0,007**	**7,175894**	**0,028**	**5,036505**	**0,004**	**7,461149**	**0,001**	**3,979773**	**0,001**
K	**5,866805**	**0,001**	**15,00132**	**0,001**	**7,582277**	**0,001**	**6,644182**	**0,001**	**8,571663**	**0,024**	**7,893713**	**0,001**	**13,23912**	**0,001**	**6,081373**	**0,001**
Mg	**3,112928**	**0,02**	**8,004556**	**0,001**	**4,531351**	**0,002**	2,461456	0,242	2,943056	0,349	3,547961	0,079	**6,948009**	**0,001**	**3,896155**	**0,005**
Ca	**3,666415**	**0,002**	**9,17133**	**0,001**	**5,266232**	**0,001**	**3,663625**	**0,046**	**12,80773**	**0,003**	**4,321875**	**0,023**	**4,366662**	**0,022**	**4,235815**	**0,006**
Moisture	**4,67853**	**0,004**	**8,044106**	**0,003**	**6,405954**	**0,001**	**6,947136**	**0,001**	**12,63784**	**0,003**	**5,948845**	**0,003**	**7,594267**	**0,001**	**4,970568**	**0,001**
Sand	**4,532691**	**0,001**	**8,680289**	**0,002**	**4,309763**	**0,001**	**5,811079**	**0,001**	**16,16077**	**0,001**	3,766129	0,057	**6,594855**	**0,001**	**3,606419**	**0,005**
Coarse silt	**6,179845**	**0,001**	**12,02387**	**0,001**	**7,633752**	**0,001**	**5,036897**	**0,006**	5,600715	0,098	2,904703	0,159	**8,224203**	**0,001**	**6,009175**	**0,001**
Fine silt	**4,274347**	**0,001**	**8,61254**	**0,002**	**3,869117**	**0,007**	**5,581207**	**0,005**	**18,0427**	**0,001**	**3,907579**	**0,045**	**6,262779**	**0,003**	**3,333163**	**0,011**
Clay	**4,42941**	**0,001**	**9,976778**	**0,001**	**4,285861**	**0,002**	**3,893781**	**0,039**	**23,18556**	**0,001**	2,911874	0,157	**3,882885**	**0,034**	**5,34241**	**0,001**

Supplementary material

Table 7. Proportion of variation in fungal community composition, at the level of the most abundant phyla explained by soil physicochemical parameters added sequentially (first to the last) in a model, depending on their independent influence in the variance as reported in Table S8.

**Ascomycota**			**Basidiomycota**			**Chytridiomycota**			**Mortierellomycota**		
Variable	Variance	p	Variable	Variance	p	Variable	Variance	p	Variable	Variance	p
**pH**	**8,619**	**0,0001**	**pH**	**10,977**	**0,0001**	**Coarse silt**	**13,951**	**0,0001**	**Moisture**	**22,504**	**0,0001**
**C**	**3,282**	**0,0015**	**Coarse silt**	**5,886**	**0,0002**	C	1,819	0,1383	**pH**	**11,194**	**0,0001**
**K**	**4,07**	**0,0002**	C	1,569	0,2418	N	1,181	0,5024	Coarse silt	2,224	0,084
**Coarse silt**	**4,189**	**0,0001**	**K**	**2,507**	**0,0417**	**K**	**5,366**	**0,0001**	**C**	**3,828**	**0,0135**
**Na**	**3,538**	**0,0003**	N	1,144	0,4901	**pH**	**4,107**	**0,0006**	**Ca**	**6,615**	**0,0007**
**Sand**	**4,183**	**0,0002**	**Sand**	**3,783**	**0,005**	**Sand**	**2,951**	**0,0113**	**CEC**	**6,279**	**0,0009**
**Fine silt**	**2,153**	**0,0196**	**Fine silt**	**3,135**	**0,0148**	**Moisture**	**5,232**	**0,0001**	**K**	**3,436**	**0,0158**
**Moisture**	**2,56**	**0,0062**	**Clay**	**4,751**	**0,002**	**Fine silt**	**2,874**	**0,0101**	Mg	2,414	0,0677
**CEC**	**7,104**	**0,0001**	Moisture	2,239	0,0771	**Clay**	**5,085**	**0,0002**	Clay	1,182	0,3037
N	1,4	0,181	**Na**	**3,564**	**0,0063**	**Na**	**2,079**	**0,0709**	Residuals	40,325	
**Clay**	**4,119**	**0,0001**	Residuals	60,446		Ca	1,36	0,3666			
**Ca**	**3,725**	**0,0001**				Residuals	53,996				
**Mg**	**2,337**	**0,0096**									
C/N ratio	1,693	0,0775									
Residuals	47,028										

Supplementary material

Table 8. Proportion of variation in fungal community composition, at the level of the most abundant phyla explained by soil physicochemical parameters calculated independently. Variance was calculated with permutational multivariate analysis of variance, based on Hellinger-transformed fungal community matrix. Significant variables were included in the final model for each fungal group (Table S7).

**Soil parameters**	**Ascomycota**		**Basidiomycota**		**Chytridiomycota**		**Mortierellomycota**	
	variance	p	variance	p	variance	p	variance	p
C	**7,312497**	**0,001**	**8,520743**	**0,001**	**11,02**	**0,001**	**17,29589**	**0,001**
N	**3,862713**	**0,011**	**6,750396**	**0,002**	**9,704722**	**0,001**	4,085636	0,107
C/N ratio	**2,925393**	**0,043**	2,689776	0,118	2,305141	0,235	3,729903	0,111
CEC	**3,893956**	**0,008**	2,526748	0,138	2,222136	0,266	**11,98343**	**0,001**
pH	**8,619303**	**0,001**	**10,97692**	**0,001**	**9,002176**	**0,001**	**19,76974**	**0,001**
Na	**5,635338**	**0,001**	**3,480439**	**0,042**	**3,864379**	**0,004**	4,295528	0,098
K	**6,852694**	**0,001**	**7,487733**	**0,001**	**9,081082**	**0,001**	**7,948711**	**0,006**
Mg	**2,998464**	**0,047**	3,323891	0,061	1,781143	0,479	**7,677108**	**0,017**
Ca	**3,451749**	**0,019**	2,941764	0,09	**3,661548**	**0,022**	**14,92539**	**0,001**
Moisture	**4,509571**	**0,005**	**4,222031**	**0,02**	**7,420497**	**0,001**	**22,50417**	**0,001**
Sand	**5,118134**	**0,002**	**5,614984**	**0,003**	**7,826468**	**0,001**	3,712676	0,122
Coarse silt	**6,01173**	**0,001**	**9,80352**	**0,001**	**13,95099**	**0,001**	**17,32184**	**0,001**
Fine silt	**4,924574**	**0,002**	**4,9461**	**0,005**	**6,423345**	**0,001**	1,593523	0,499
Clay	**3,780879**	**0,011**	**4,434432**	**0,011**	**4,073938**	**0,02**	**6,231061**	**0,036**
